# Discerning the Antimicrobial Resistance, Virulence, and Phylogenetic Relatedness of *Salmonella* Isolates Across the Human, Poultry, and Food Materials Sources in Malaysia

**DOI:** 10.3389/fmicb.2021.652642

**Published:** 2021-08-31

**Authors:** Zunita Zakaria, Latiffah Hassan, Norazah Ahmad, Suraya Amir Husin, Rohaya Mohd Ali, Zawiyah Sharif, Norfitriah Mohamed Sohaimi, Bashiru Garba

**Affiliations:** ^1^Institute of Bioscience, Universiti Putra Malaysia, Serdang, Malaysia; ^2^Department of Veterinary Pathology and Microbiology, Faculty of Veterinary Medicine, Universiti Putra Malaysia, Serdang, Malaysia; ^3^Department of Veterinary Laboratory Diagnostics, Faculty of Veterinary Medicine, Universiti Putra Malaysia, Serdang, Malaysia; ^4^Infectious Diseases Research Centre, Institute for Medical Research, National Institutes of Health, Selangor, Malaysia; ^5^Medical Development Division, Ministry of Health, Putrajaya, Malaysia; ^6^Diagnostic and Quality Assurance Division, Department of Veterinary Services, Ministry of Agriculture and Agro-Based Industry, Putrajaya, Malaysia; ^7^Food Safety and Quality Division, Ministry of Health, Selangor, Malaysia; ^8^Department of Veterinary Public Health and Preventive Medicine, Faculty of Veterinary Medicine, Usmanu Danfodiyo University, Sokoto, Nigeria

**Keywords:** *Salmonella*, whole-genome sequencing, antimicrobial resistance, phylogenetic studies, virulence gene profile, foodborne infections, *Salmonella enteritidis*

## Abstract

*Salmonella enterica* subspecies *enterica* serovar Enteritidis is one of the major foodborne zoonotic pathogens globally. It has significantly impacted human health and global trade. In this investigation, whole-genome sequencing was employed to determine the antimicrobial resistance (AMR) pattern of a collection of *Salmonella* Enteritidis isolated from humans, poultry, and food sources. The study also investigated the virulence genes profile of the isolates as well as the phylogenetic relationships among strains. Illumina NextSeq technology was used to sequence the genome of 82 *Salmonella* Enteritidis strains isolated over 3 years (2016–2018) in Peninsular Malaysia. The pattern of resistance showed that tetracycline had the highest frequency (37/82, 45.12%), and isolates from food samples showed the highest rate of 9/18 (50.00%), followed by human 17/35 (48.57%) and then poultry 11/29 (37.93%). The second drug with the highest resistance rate is ampicillin with 5/29 (17.24%) for poultry, 4/35 (11.43%) for human, and 0/18 (0.00%) for food isolates respectively. Similarly, a total of 19 antimicrobial resistance (AMR) genes corresponding to the nine drugs used in the disc diffusion assay were evaluated from the whole genome sequence data. The aminoglycoside resistance gene *aac(6′)-ly* was detected in 79 of the 82 isolates (96.34%). While the phylogenetic analysis revealed distinct lineages isolated, the three sources indicating possible cross-contamination. In conclusion, the results showed that the genomic profile of *Salmonella* Enteritidis isolated from humans, poultry, and food samples share genetic traits, hence the need to institute measures at controlling the continuous spread of these resistant pathogens.

## Introduction

*Salmonella* is a very important zoonotic pathogen that has been reported to cause over 200 million human clinical infections with an estimated mortality of 3 million annually (Coburn et al., 2007; Graham et al., 2018; Moussa et al., 2021). Humans easily acquire infection from contact with animal or environmental reservoirs. The emergence of antimicrobial-resistant *Salmonella enterica* subspecies *enterica* serovar Enteritidis constitutes a serious global health problem (Pan et al., 2018). This phenomenon is believed to be due to the unregulated use and abuse of antimicrobials especially veterinary drugs including the World Health Organization’s critically important antimicrobials for prophylaxis, during the management of diseases or as growth promoters (Azmi et al., 2018; Sharma et al., 2018; Yu et al., 2021). *S.* Enteritidis is one of the most important serovars shared between humans and animals in most parts of the world (Mohan et al., 2019). It is notorious for its ability to colonize livestock; wild animals as well as the reproductive system of chickens where they persist without any apparent clinical manifestation, thus continuously contaminate eggs and the immediate environment (Campioni et al., 2018; Abdulhaleem et al., 2019). The sustained and undetected presence of these bacteria in food-producing animals makes it possible to cause prolonged epidemics globally, especially where the consumption of poorly cooked poultry egg and meat is common (Salihu et al., 2015; Judd et al., 2019).

*Salmonella* Enteritidis is one of the major cause of invasive salmonellosis globally, particularly among malnourished children and adults with debilitating illnesses (Stanaway et al., 2019). Population-based estimates of the burden of enteric *Salmonella* infection in Southeast Asia range from 2.2 to 10.7 cases/100,000 people annually (Whistler et al., 2018). Similarly, *S.* Enteritidis is reported to account for 38% of all clinical cases in the Southeast Asia region (Eng et al., 2015). Recent investigations in Thailand on the causes of bacteremia discovered that *S*. Enteritidis accounts for 51.6% of all cases of invasive non-typhoidal *Salmonella* infections (Phu Huong Lan et al., 2016; Whistler et al., 2018). In a related investigation to determine the major food pathogens isolated in food materials in China, *Salmonella*, alongside *Vibrio parahaemolyticus*, and *Campylobacter* were among the most prevalent (Paudyal et al., 2018). These observations points to the continued risk humans face from these infectious disease pathogens, particularly where they exhibit antimicrobial resistance (AMR).

Gastro-enteric infection as a result of *S.* Enteritidis has persisted in Malaysia and continues to be a serious public health problem (Thung et al., 2016; Zakaria et al., 2020). The incidence rate has steadily increased in recent years, and this may be attributed to the unhygienic mode of handling and processing of food, especially poultry and poultry products (Thung et al., 2018; Mohan et al., 2019). Although considerable measures to improve food safety has been put in place by the relevant public health authorities, pockets of outbreaks involving large populations continue to be recorded (Balkis et al., 2017; Packierisamy et al., 2018). Other possible reasons why *S*. Enteritidis continues to be a health burden in Malaysia include the proliferation of rodent reservoirs in poultry farms, households, and public food restaurants, evolutionary selection, and the emergence of antimicrobial-resistance among other evolutionary traits (Porwollik et al., 2005; Betancor et al., 2009; Campioni et al., 2018; Azmi et al., 2021). In Malaysia like many parts of the world, it is believed that resistant *S*. Enteritidis had been introduced into the country through the importation of poultry meats and poultry products from other endemic countries (Elemile et al., 2019). In light of these explanations, and the need to understand the phenotypic and genomic diversity of circulating *S.* Enteritidis strains in Malaysia, a Whole-genome sequencing approach was used to assess the phylogenetic relationships, virulence factor determinants, and the antimicrobial-resistance profile of a collection of *S*. Enteritidis isolated from the Central region of Peninsular Malaysia from 2016 to 2018, with a view to understand the genetic relationship of this important pathogen across different sources (human clinical samples, live birds, and chicken meat).

## Materials and Methods

### *Salmonella* Isolates Selection

The 82 *Salmonella* isolates used for this study were obtained from government diagnostic laboratories operating within Peninsular Malaysia as part of an ongoing National Antimicrobial Surveillance Program. The isolates were collected from multiple sources, including humans, poultry, and food. *Salmonella* was isolated at various time points from 2016 to 2018. The human *Salmonella* isolates (*n* = 35) were obtained from blood and stool samples collected from clinical cases and provided by the Bacteriology Unit, Institute for Medical Research, Ministry of Health Malaysia. Poultry samples (29) originated from fecal samples from commercial poultry farms provided by the Antimicrobial Surveillance Section, Department of Veterinary Services, Ministry of Agriculture Malaysia. At the same time, the food isolates were obtained from ready to eat food from restaurants and chicken meats from wet markets (18) and were provided by the Food Safety and Quality Division, Ministry of Health Malaysia. All the samples were stored in nutrient agar slants until required for further characterization.

### Serotype Prediction and Evaluation of Phenotypic AMR

Traditional serotype determination, according to the Kauffmann White Scheme and the *in silico* serotype prediction based on the raw reads and genome assemblies, were conducted using SeqSero2 (SeqSero2 v1.1.0) pipeline (Zhang et al., 2019). While the Kauffmann White Scheme determines *Salmonella* serovars using standard agglutination (SSI Diagnostica *Salmonella* Sero-quick ID kit, UC Bioscience Sdn, Bhd) method according to their antigenic formula (O and H antigens), the SeqSero 2 software uses a k-mer based algorithm to predict serovars from raw reads which ensures improved serotype prediction from draft genome assemblies (Diep et al., 2019; Zhang et al., 2019).

Similarly, all the *Salmonella* isolates serotyped using the traditional Kauffmann White Scheme and then subjected to phenotypic antimicrobial susceptibility test using the Kirby Bauer Disc Diffusion method. A panel of nine (9) antimicrobials purchased from Oxoid (Thermo Scientific Microbiology Sdn Bhd)including ampicillin (Amp-R < 13 mm), chloramphenicol (C-R < 12 mm), gentamicin (CN-R < 12 mm), streptomycin (S-R < 11 mm), sulfamethazine/trimethoprim (SXT-R < 10 mm), tetracycline (TE-R < 11 mm), ceftiofur (EFT-R < 10 mm), cefotaxime (Ctx-R < 22 mm), and ciprofloxacin (CIP-R < 15 mm) was used (Wayne, 2019). *Escherichia coli* ATCC25922 was used as internal quality control. The *Salmonella* isolates with the zone of inhibition within the intermediate range were considered to be susceptible to avoid overestimation of resistance.

### Genome Library Preparation, Sequence Assembly, and Annotation

About 2 ml of an overnight culture (at 37°C) of the *Salmonella* isolates (*n* = 82) at on Luria-Bertani (L.B.) agar was pelleted by centrifugation at 5000 × *g* for 10 min. The genomic DNA was extracted using QIAamp DNA Mini Kit (Qiagen), and the purity and concentration were determined using the NanoDrop spectrophotometer (Thermo Fisher Scientific). The Nextera^TM^ DNA Flex Library Prep Kit was employed for the preparation of the genomic libraries. The whole-genome sequencing was performed on the NextSeq 550 System (Illumina, United States). Sequencing reads obtained from the Illumina NextSeq sequencer were scanned for adapter sequence and low-quality sequence using BBDuk (BBTools version 36), where adapter trimming, quality trimming, contaminant filtering, and read length filtering was done, and part of the reads containing poor quality sequence were removed. The good quality sequencing reads were then assembled using SPAdes (SPAdes version 3.9.0) to obtain contigs (Bankevich et al., 2012).

Due to the different sequence profiles, the gene annotation was achieved by predicting the rRNA genes using RNAmmer (Lagesen et al., 2007), while the tRNA genes were predicted using ARAGORN (Laslett and Canback, 2004). The protein-coding genes were first predicted using Prodigal (Hyatt et al., 2010), and the predicted sequences were used to predict their function by using BLAST (Camacho et al., 2009) and HMMER (Eddy, 1998) to search against various sequence or domain databases.

### Comparative Analysis

All the assembled contig sequences from the 82 *Salmonella* isolates as well as representative reference genomes of closely related *Salmonella* species (CP019177.1, CP036165.1, CP036166.1, CP022500.1, CP037917.1, CP019183.1, CP022489.1, CP003278.1, AE006468.2, NC_003197.2, and CP014996.1) obtained from the NCBI database were used to infer the relationship, while *Salmonella bongori* (NC_015761.1) and *E. coli* (NC_000913.3) were used as outgroup controls. All the sequences were subjected to comparative studies using the EPInod pipeline developed by BioEasy Sdn Bhd. The software package is capable of evaluating the sequences for average nucleotide identity (ANI), multi-locus sequence typing (MLST), Single Nucleotide Polymorphism (SNP)-based phylogenomics estimation, pan-genome gene conservation analysis, virulence factor (V.F.) detection, and AMR gene detection.

Whole-genome was utilized to deduce the genetic relationship among isolates from different species. The identification of pan-genome SNPs and phylogenetic analysis was made using the kSNP3.0 program, as described by Gardner et al. (2015). The FASTA input file of the target genomes for SNPs discovery was first input, and k, which represents the length of the flanking sequence, including the SNPs, was specified. Finally, SNPs positions in the finished genomes were found by matching with MUMmer. The phylogenetic tree was inferred using all genome SNPs aligned multiple FASTA files with MEGA7 (Kumar et al., 2016). The maximum-likelihood phylogenetic tree of all SNPs loci was generated using the Jukes-Cantor/GTI model with calculated SH-like branch support (1,000 iterations), not bootstrapping. This is because SH-like branch support is fast and efficient, and is commonly used as the default method of choice for phylogenetic software.

Similarly, the genome Virulence Factor analysis to determine the major virulence factors of the characterized bacterial pathogen was achieved using the virulence factor database (VFDB), which is an integrated and comprehensive online resource for curating information about virulence factors of bacterial pathogens (Chen et al., 2016). The NCBI BLAST tool was used to scan predicted CDS (nucleotides) against VFDB for virulence factors, which represent the measure of the pathogenicity of the isolates. The virulence factors analysis was done in two sets, where set A includes genes with experimentally verified V.F. only while set B covers all genes related to known and predicted V.Fs in the V.F database. Finally, the Genome Antibiotic Resistance to identify the AMR profile of the isolates was streamlined via the abricate program against ResFinder and the Comprehensive Antibiotic Resistance Database (CARD) to screen contigs for AMR.

### Correlation Between the Phenotypic and Genotypic AMR Profiles

The antimicrobial susceptibility pattern of the isolates was determined with the Kirby Bauer disk diffusion test, as mentioned earlier. The AMR pattern of the isolates was correlated with its known corresponding resistance gene for each of the strains detected by the WGS analysis, and the percentage correlation was calculated. This was done by counting the genotypic results and dividing the total by the number of isolates that exhibited phenotypic resistance to determine the sensitivity. While the specificity was calculated by dividing the number of isolates that showed genotypic susceptibility by the total number of isolates showing phenotypic susceptibility (Trevethan, 2017). The percentages of positive predictive values (PPVs) and negative predictive values (NPVs) were also calculated as described by Trevethan (2017).

### Statistical Analysis

The association between AMR determinants and virulence genes is calculated using the Chi-square test, and the *P*-value level <0.05 is considered significant. Similarly, the correlation between the phenotypic and genetic AMR was also deduced. A one-way analysis of variance (ANOVA) was also conducted to determine if there is significant difference between the means of the three hosts (Human, Poultry and Food), followed by a *post hoc* for multiple comparisons.

## Results

The Kauffmann White Scheme serotyping tests indicated that all the isolates from the three different sources (human, poultry, and food) belonged to the *S*. Enteritidis serogroup. However, the overall results suggested that SeqSero2 (SeqSero2 v1.1.0) improved the serotype prediction compared to the Kauffmann White Scheme serotyping scheme. Although 93% of the serotype prediction according to the Kauffmann White Scheme was concordant with the *in silico* WGS prediction, six isolates were found to conflict as they were predicted to be Brancaster (S6-human), Mbandaka (S18-human), Ohio (S72-poultry), Weltevreden (S77-poultry), and Kentucky (S81-poultry), while S63 (poultry) was found to be either Albany or Duesseldorf because they share the same antigenic formula (8: z4, z24). Additionally, the SeqSero2 also identified one unique isolate with antigenic formula I 4:b:- (S87-poultry), which is not listed in the Kauffmann White Scheme. The antimicrobial susceptibility assay, according to the Clinical and Laboratory Standards Institute (CLSI) standards (Patel et al., 2016), showed that thirty (30) isolates were resistant to at least one of the tested antimicrobial drugs, while twenty-four (24) were resistant to multiple drugs tested (MDR: resistance to three or more antimicrobial classes), and the remaining isolates were all susceptible to the tested antimicrobials. However, only one isolate showed resistance to each of ceftiofur (1 human isolate), cefotaxime (1 human isolate), sulfamethazine/trimethoprim (1 food isolate), gentamicin (1 poultry isolate), and chloramphenicol (1 human isolate), while none of the isolates were resistant to ciprofloxacin. The phenotypic and genotypic antimicrobial susceptibility results are summarized in Tables 1, 2.

**TABLE 1:** Distribution of phenotypic antimicrobial resistance (AMR) profile among *S*. Enteritidis isolates.

Isolates	Antimicrobial Resistance (%)								
	
	AMP	C	CN	S	SXT	TE	EFT	CTX	CIP
Poultry	5/29 (17.24%)	0/29 (0.00%)	1/29 (3.45%)	1/29 (3.45%)	0/29 (0.00%)	11/29 (37.93%)	0/29 (0.00%)	0/29 (0.00%)	0/29 (0.00%)
Food	0/18 (0.00%)	0/18 (0.00%)	0/18 (0.00%)	0/18 (0.00%)	1/18 (5.56%)	9/18 (50.00%)	0/18 (0.00%)	0/18 (0.00%)	0/18 (0.00%)
Human	4/35 (11.43%)	1/35 (2.86%)	0/35 (0.00%)	1/35 (2.86%)	0/35 (0.00%)	17/35 (48.57%)	1/35 (2.86%)	1/35 (2.86%)	0/35 (0.00%)
Overall	9 (10.98%)	1 (1.22%)	1 (1.22%)	2 (2.44%)	1 (1.22%)	37 (45.12%)	1 (1.22%)	1 (1.22%)	0 (0.00%)

*The antimicrobial susceptibility/resistance was determined based zone of inhibition according to the CLSI standard. *Ampicillin* (*AMP*), *chloramphenicol* (*C*), *gentamicin* (*C.N.*), *streptomycin* (*S*), *sulfamethazine/trimethoprim* (*SXT*), *tetracycline* (*T.E.*), *ceftiofur* (*EFT*), *cefotaxime* (*CTX*), and *ciprofloxacin* (*CIP*).*

**TABLE 2:** Distribution of genotypic AMR genes among *S*. Enteritidis isolates.

Source of isolate	Antimicrobial Resistance (%)								
	
	AMP (*TEM 33*; *TEM 4*)	C (*floR*)	CN (*aac*(*6*′)-*ly*; *aadA*)	S (*strA; strB*)	SXT (*sul1; sul2; dfrA14; dfrA15*)	TE (*tetA; tetC*)	EFT (*blaCMY-2; blaTEM-1*)	CTX (bla*-TEM*, bla*-CTX-M*)	CIP (*qnrS1; qnrD1*)
Poultry	6/29 (20.69%)	−/29 (0.00%)	28/29 (96.55%)	1/29 (3.45%)	−/29 (0.00%)	10/29 (34.48%)	−/29 (0.00%)	−/29 (0.00%)	1/29 (3.45%)
Food	−/18 (0.00%)	−/18 (0.00%)	17/18 (94.44%)	−/18 (0.00%)	1/18 (5.56%)	9/18 (50.00%)	−/18 (0.00%)	−/18 (0.00%)	−/18 (0.00%)
Human	3/35 (8.57%)	1/35 (2.86%)	34/35 (97.14%)	−/35 (0.00%)	2/35 (5.71%)	19/35 (54.29%)	−/35 (0.00%)	1/35 (2.86%)	2/35 (5.71%)
Overall	9 (10.98%)	1 (1.22%)	79 (96.34%)	1 (1.22%)	3 (3.66%)	38 (46.34%)	0 (0.00%)	1 (1.22%)	3 (3.66%)

*The table presents multiple genes that confer resistance against the antimicrobial drugs used for the antimicrobial susceptibility test (AST) analysis. *Ampicillin* (*Amp*), *chloramphenicol* (*C*), *gentamicin* (*C.N.*), *streptomycin* (*S*), *sulfamethazine/trimethoprim* (*SXT*), *tetracycline* (*T.E.*), *ceftiofur* (*EFT*), *cefotaxime* (*CTX*), and *ciprofloxacin* (*CIP*).*

**FIGURE 1:**
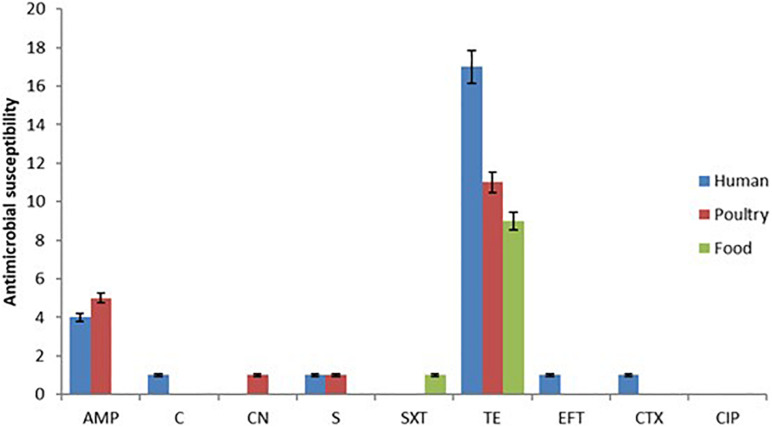
Showing the antimicrobial susceptibility test results based on the Kirby Bauer disk diffusion method indicating high resistance to tetracycline and ampicillin.

**FIGURE 2:**
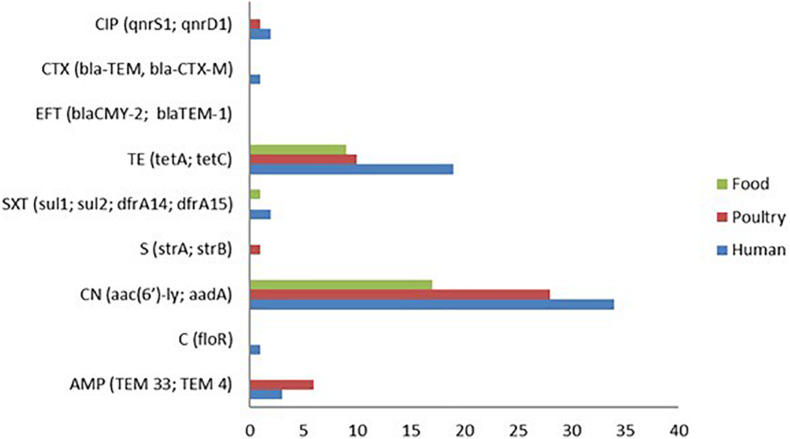
Genotypic antimicrobial resistance (AMR) patterns of *S.* Enteritidis isolates showing aminoglycoside resistance genes (*aac (6′)-ly*) and *aadA* as the predominant resistance genes detected followed by tetracycline (*tetA and tetC*) genes.

The pattern of resistance showed that tetracycline had the highest frequency (37/82, 45.12%), and isolates from human samples showed the highest rate with 17/35 followed by poultry 11/29 and then food 9/18 (Figure 1). The second drug with the highest resistance rate is ampicillin with 4/35 (11.43%) for human, 5/29 (17.24%) for poultry, and 0/18 (0.00%) for food isolates respectively (Figure 2). However, the chi-square statistic to check for any association between the resistances observed and the source of the samples (human, poultry, or food) was found to be 1.772, and the *p*-value was 0.412. This signifies that the association is not significant at *p* < 0.05. Similarly, no statistically significant difference was observed based on the ANOVA with a *p*-value of 0.704 (*p* > 0.05).

A total of 19 antimicrobial resistance (A.R) genes corresponding to the nine drugs used in the disc diffusion assay were evaluated from the whole genome sequence data. The aminoglycoside resistance gene *aac(6′)-ly* was detected in 79 of the 82 isolates (96.34%). This observation is in contrast to the phenotypic analysis, which showed that only one gentamicin-resistant isolate (1.22%) was found among all the isolates. Although, the detection of the tetracycline-resistant genes (*tetA*, *tetC*, and *tetD*) 38/82 (46.34%) was found to correspond with the phenotypic result for tetracycline 37/82 (45.12%), albeit with the genotypic detection being higher by one isolate (Figure 2). However, the predominant tetracycline resistance encoding gene was *tetC*, with only one isolate possessing the *tetA* gene.

Ten (10) isolates were resistant to beta-lactams (10.98%), and the two genes encoding beta-lactamases identified in these isolates were *TEM33* (10/82), and *TEM4* (10/82). Not a single isolate was found to show resistance to the quinolone ciprofloxacin based on the disc diffusion method. However, the genes *qnrS*, and *qnrD1* were detected from two human isolates and one poultry isolate. Other resistance genes identified by the whole-genome AMR analysis are *dfr*A14 (3/82), *dfr*A15 (3/82), and *sul2* (2/82) for trimethoprim and sulfonamide resistance as well as one *floR* gene (1/82) that codes for chloramphenicol resistance.

### Correlation Between AMR Based on Kirby Bauer Disc Diffusion Test and Whole-Genome Sequence AMR Analysis

The data generated from the whole-genome sequence AMR analysis was correlated with the phenotypic AMR to evaluate the ability of the whole-genome sequence data to predict the phenotypic AMR profiles obtained by the disc diffusion method. The predominant phenotypic AMR resistance observed was resistance against tetracycline (45.12%) and ampicillin (10.98%). However, the analysis did not include ciprofloxacin because none of the isolates studied showed phenotypic resistance to ciprofloxacin. Generally, a strong correlation between the phenotypic resistance by disc diffusion and the AMR gene prediction by WGS was observed, except for gentamicin, which was discordant (Table 3).

**TABLE 3:** Correlation between AMR phenotype by Kirby Bauer method and genotypic AMR WGS analysis for *S.* Enteritidis isolates (*n* = 82).

Antimicrobials	Resistant isolates		Susceptible isolates		Sensitivity (%)	Specificity (%)	PPV (%)	NPV (%)
	Genotypic	Phenotypic	Genotypic	Phenotypic				
AMP	9	9	73	73	10.98	89.02	50.00	50.00
C	1	1	81	81	1.22	98.78	50.00	50.00
CN	79	1	3	81	96.34	98.78	98.75	96.43
S	1	2	81	80	1.22	97.56	33.33	49.69
SXT	3	1	79	81	3.66	98.78	75.00	50.63
TE	38	37	44	43	46.34	53.75	50.67	49.43
EFT	0	1	82	81	0.00	98.78	0.00	49.69
CTX	1	1	81	81	1.22	98.78	50.00	50.00
Total					20.12	91.78	50.97	55.73

*Ampicillin (AMP), Chloramphenicol (C), Gentamicin (C.N.), Streptomycin (S), sulfamethazine/trimethoprim (SXT), Tetracycline (T.E.), Ceftiofur (EFT), Cefotaxime (CTX), and Ciprofloxacin (CIP), PPV-Positive Predictive Value, NPV-Negative Predictive Value.*

The overall sensitivity for the WGS based AMR prediction across all the antimicrobial agents evaluated was 50.97%, while the specificity was 55.73%. The post-test probability of the AMR prediction, which measures how well the technique predicts the resistance accurately, was 20.12%, while the NPV was 91.78%.

### Analysis of *S.* Enteritidis Virulence Determinants

For this article, only those virulence genes that are associated with experimentally verified virulence factors were considered. The virulence factors included bacterial toxins, cell surface proteins that mediate the attachment, cell surface carbohydrates, and proteins that protect the bacterium and hydrolytic enzymes that may contribute to the pathogenicity of the bacteria. These genes were analyzed using the BLAST program against the VFDB. The parameters used were the Percent identity cut-off (50), Percent query coverage per high-scoring segment pair (hsp) (50), and the number of aligned sequences to keep (Chen et al., 2016). A total of 121 virulence genes were detected by the BLAST search against VFDB. The virulence-associated determinants identified in this study include a group of transport, adhesion and some effector proteins such as; *Salmonella* virulence plasmid determinant protein, Mg^2+^ transport protein, Intimin-like protein, resistance to compliment killing protein, type III secretion system effector and plasmid-encoded fimbria protein, among other (Supplementary Table 1).

The virulence genes identified in this study are predominantly genes belonging to the type III secretion system (T3SS), which is encoded by the *Salmonella* pathogenicity sequence 1 (SPI-1) and *Salmonella* pathogenicity sequence 2 (SPI-2). The type III SPI 1 genes include; system effector (*SteA* and *SteB*), type III secretion system accessory cytosolic protein (OrgA and OrgC), type III secretion system regulatory protein (InvA-InvJ), type III secretion system export apparatus switch protein (SpaO-SpaS), and type III secretion system hydrophilic translocator, and pore protein (SipA-SipD) (Supplementary Table 1). These genes were detected in all the isolates from the human, poultry, and food samples. Similarly, SPI-2 system effector (SopB, SopD2, and SlrP) chaperone protein-coding (sseB-sseC) *SteC, SseK1, SifB, SseK2*, and type III secretion system gatekeeper (*SsaH-SsaL*) were among the genes detected in all the isolates except S63 isolate from poultry swab.

In addition to the genes within the pathogenicity sequences, virulence plasmid (plasmid-encoded fimbriae chaperone protein PefD), ion acquisition (ferrienterobactin outer membrane transporter), fimbriae (long polar fimbrial chaperone protein, type I fimbriae adaptor protein FimF), as well as the flagella and flagellin genes (flagellar motor protein MotA) were all identified. Within the fimbrial adherence determinants, are genes that codes for the curli fimbriae and curli assembly protein CsgC that mediate the binding to various serum and tissues matrix proteins (Thomas et al., 2017). Other important virulence determinants detected from most of the isolates after the BLAST analysis within the VFDB software are; the antimicrobial peptide resistance protein Mig-14, Mg^2+^ transport protein MgtBC, and the *Salmonella* plasmid virulence which is responsible for the regulation of spv operon.

### Phylogenomics of *S.* Enteritidis

Phylogenetic analysis for the 82 genome sequence (WGS data attached as a Supplementary Document 2 – https://submit.ncbi.nlm.nih.gov/wgs_common/report/SUB8477823) was conducted using the maximum likelihood method. The analysis included the sequence from the 35 human isolates, 18 isolates from food, and 31 isolates obtained from poultry cloacal swabs and an *S.* Brancaster, *S.* Ohio, *S*. Mbandaka, *S.* Kentucky, *S.* Weltevreden, *S.* Typhimurium, *S.* Bongori, and *E. coli* K12 control genome. Two bifurcating clades and one monophyletic clade from the ancestral node were confirmed from the phylogenetic tree. The rooted *S.* Enteritidis tree indicated multiple bifurcating patterns with varying levels of diversity and placed both *S.* Enteritidis isolates from humans, poultry, and food samples into distinct monophyletic clades, suggesting that the isolates evolved into distinct evolutionary lineages from their common phylogenetic ancestor. The ancestral node branched into two with the *E. coli* (clade A) outgroup diverging away from the *Salmonella* group (clade B). Subsequently, clade B bifurcated into sub-clade B1 occupied by *S.* Bongori and B2 (*S. enterica*), which later splits into multiple clusters with variable genetic properties compared to the reference genomes.

The phylogenetic analysis revealed that sub-clade B2-I contained S6 and S18 from human clinical samples, which clustered with *S*. Brancaster and *S*. Mbandaka, respectively. While S63, S72, S77, and S81 clustered with *S.* Albany, *S.* Ohio, *S.* Weltevreden, and *S*. Kentucky, respectively (sub-clade B2-I). The clustering of these isolates with the reference genome conforms with the SeqSero serotype allocation. Important to note that while the SeqSero analysis identified that S63 shared the same antigenic formula with *S.* Albany and *S.* Duesseldorf, the phylogenetic tree showed it is more genetically related with *S.* Albany rather than *S*. Duesseldorf. *S*. Typhi, which is typhoidal *Salmonella*, was found to share some genetic relationship with *S.* Ohio, *S.* Enteritidis, and *S.* Typhimurium having branched from the same bifurcation. Sub-clade B2-II, on the other which contained the largest convergence of the isolates, implies close genetic relation across the different sources.

## Discussion

*Salmonella* serotyping using the traditional phenotypic method was the first analysis conducted in this study because of its importance in the characterization of *Salmonella*, especially during epidemiological investigations and surveillance programs (Diep et al., 2019). However, because of the ease and superior predictability of WGS based *in silico* methods, SeqSero2 was used to confirm the phenotypic serotyping (Zhang et al., 2019). The results show that the SeqSero2 correctly identified *S*. Enteritidis in concordance with the Kauffmann White Scheme result with 93% similarity. However, the SeqSero2 was able to identify six serovars other than *S*. Enteritidis (Brancaster, Mbandaka, Ohio, Weltevreden, and Kentucky), including Albany and Dusseldorf which had the same antigenic formula (I 4:b:-), while one was not available in the database. This finding agrees with earlier studies that indicated that the same antigenic formula could be shared by different subspecies (Diep et al., 2019). *In silico* WGS-based serotyping has been shown to perform better in identifying *Salmonella* serotype diversity compared to the traditional method and has become popular among molecular epidemiologists and public health scientists (Diep et al., 2019; Elnekave et al., 2020).

The present investigation also evaluated AMR determinants, virulence factor genes, and phylogenetic relationships of the isolates using a whole-genome sequencing approach. This is because comparative genomics studies have indicated that host specificity (humans, animals, birds, and environment) is a very important factor driving the evolution of new lineages of enteric *Salmonella* species (Tasmin et al., 2017; Deblais et al., 2018). Secondly, the choice of WGS is because its application permits a broader inference of pathogen characterization, including the prediction of antibiotic resistance and virulence determinants from the sequence (Gupta et al., 2019). The 82 *S.* Enteritidis, *S*. Brancaster, *S.* Mbandaka, *S*. Albany, *S*. Ohio, *S.* Weltevreden, and *S.* Kentucky isolates identified in this study were cultured from multiple sources, including human clinical samples (blood and feces), poultry (cloacal swab), and food (chicken meat). Upon obtaining the whole genome sequence, EPInod pipeline was used for the analysis of AMR profile, virulence determinants, and the SNP-based phylogenetic analysis.

Antimicrobial usage represents a very critical factor in the emerging public health crisis due to antibiotic resistance. Its extensive application in food animal production to manage clinical infections, as prophylaxis to prevent and control livestock diseases, as well as its use as an additive in feed to enhance growth and productivity have all played roles in the emergence of resistance (Salihu et al., 2013; Wang et al., 2019; Van et al., 2020; Ma et al., 2021). In this study, tetracycline (45.12%) and ampicillin (11.43%) were identified as drugs with the highest phenotypic resistance rate. Similarly, resistance to these drugs was highest among the human isolates (17/35), followed by food isolates (9/18) and then poultry (11/29). These antimicrobials are among those that have been declared critical by the WHO and their use in animals has been restricted. This finding implies that some of these legislation is not being observed and this could lead to the loss of potency of the drugs and progressive emergenc of antimicrobial resistant pathogens. Also, 29.27% of the isolates comprising of *S*. Enteritidis, *S*. Mbandaka, *S*. Ohio, *S*. Kentucky, and *S.* Brancaster exhibited multiple resistances to 2 or more drugs. According to studies sanctioned by the Department of Veterinary Services Malaysia under the Livestock Farm Practices Scheme (SALT) program, for *Salmonella* species isolated from chicken cloacal swabs revealed that 13.5% of the *Salmonella* isolates were resistant to tetracycline, 5.4% to polymyxin and erythromycin. This is similar to the investigation conducted in China where tetracycline resistance was found to predominate among *Salmonella* isolates obtained from slaughtered pigs (Wu et al., 2021). In comparison, 2.7% were resistant to chloramphenicol and trimethoprim (Azmi et al., 2018). Despite the resistance observed in this study, the association between the resistance rate and the source of the sample as well as statistical significance based on ANOVA between their means was found not to be statistically significant (*p* > 0.05). Other drugs that were found to exhibit resistance were chloramphenicol among human isolates (2.86%), gentamicin in poultry (3.45%), sulfamethazine/trimethoprim in food (5.56%), ceftiofur and cefotaxime both in human (2.86%). In a related study conducted in the East Coast of Peninsula Malaysia, *Salmonella* isolates from broiler chicken were found to exhibit resistance to chloramphenicol (76.2%), sulfamethoxazole/trimethoprim (42.9%) (Ibrahim et al., 2021). This further highlights the crucial role of poultry and poultry products in the dissemination of resistant Salmonella pathogens to humans and other susceptible animal species.

On the other hand, the WGS analysis of AMR genes correlated significantly by identifying some genes that encode resistance to the majority of the phenotypic resistance observed. These included tetracycline, ampicillin, streptomycin, sulfamethazine/trimethoprim, cefotaxime, and ciprofloxacin resistance genes such as *tetA*, *tetC*, *TEM33*, *TEM4, floR*, *aac(6′)-ly*, *strA*, *sul1*, *dfrA14*, *dfrA15*, *blaCTX-M*, and *qnrS1*, respectively (Table 2). Although no phenotypic resistance was observed for gentamicin, the AMR genotype result showed that the gentamicin gene (*aac(6′)-ly*) was the most frequent (96.34%) with isolates from a human source having the highest percentage (97.14%). This observation goes to show WGS as an excellent tool for the accurate prediction of antimicrobial-resistant phenotype in human, animal, and environment samples (Pornsukarom et al., 2018; Liu et al., 2021). The gene *aac(6′)-ly* is one of the important chromosomal genes responsible for the enzymatic modification of aminoglycoside leading to the development of resistance (Odumosu et al., 2015; El-Badawy et al., 2017). However, it is important to note that mechanism for aminoglycoside resistance include among others decreased uptake and accumulation of the drug in the bacterial pathogen, as well as the expression of aminoglycoside modifying enzymes (AMEs) that causes inactivation of the drug (Mir et al., 2016). Even though, a strong correlation between the phenotypic resistance by disc diffusion and the AMR gene prediction by WGS was observed (sensitivity 20.12% and specificity 91.78%), the result for gentamicin was discordant (Table 3). The most common genes conferring resistance to β-lactam antibiotics in this study were *TEM 33* and *TEM 4* without corresponding phenotypic consequence. Qualitative determination of antimicrobial susceptibility based on the zone of inhibition is prone to error due to the instability of antibiotic-containing discs under varying temperature and humidity, interference of the functionality of some antimicrobials by the components of growth agar, and the possibility that an isolate may be falsely classified as susceptible (Neuert et al., 2018; Srivastava et al., 2020). This may explain the relative number of mismatches observed in the present study. Moreover, many of the resistance genes detected by the WGS algorithm are plasmid-encoded. In other words, bacterial plasmids are known to commonly cure during storage and sub-culture of the isolates, such that genes detected during sequencing might have become altered (Neuert et al., 2018). Furthermore, *Salmonella* has been shown to possess silent resistance genes, as well as variants (*aac(6′)*), which only become transcriptionally active in rare cases (Heider et al., 2009; Frye and Jackson, 2013; Adesiji et al., 2014).

**FIGURE 3:**
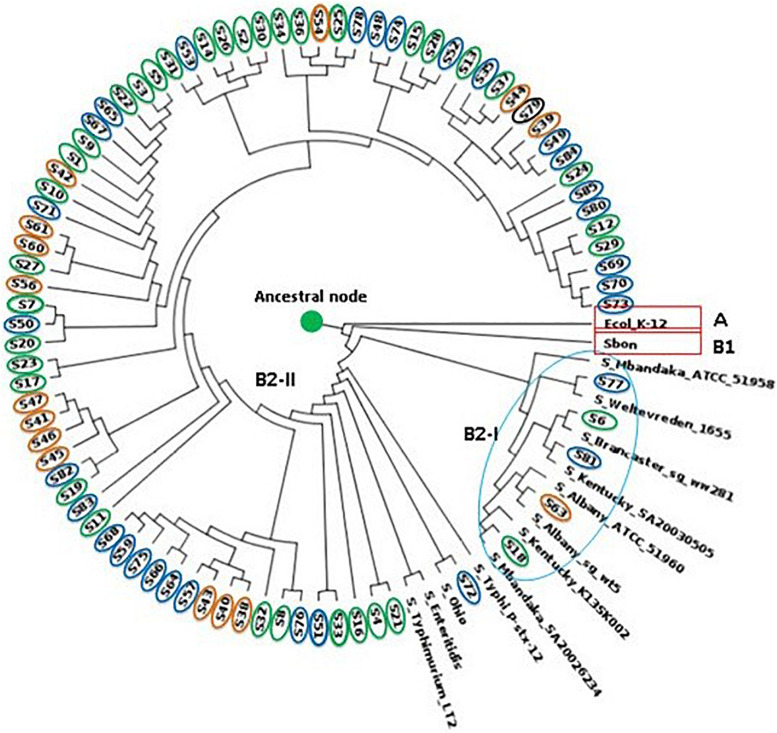
Phylogram showing maximum-likelihood phylogeny of *S.* Enteritidis calculated using aligned all genome SNPs. Human isolates circled in green, food in orange and chicken isolates in blue.

The WGS analysis revealed multiple virulence genes among the *Salmonella* isolates across the different sources (Supplementary Table 2). These genes included the type III secretion system (T3SS) located within the *Salmonella* Pathogenicity Sequence I and II, intimin-like proteins, plasmid-encoded fimbriae chaperone protein, host recognition/invasion determinants, as well as magnesium uptake, iron acquisition factors. These genes are responsible for *Salmonella* infectivity, transmission, and survival (Pornsukarom et al., 2018). The result of the present study indicates that the *S.* Enteritidis isolated from the poultry cloacal swabs and food sources possessed the same virulence genes as the human clinical isolates. Virulence factors such as adhesins, toxins, and iron ion transport systems are responsible for the adhesion of pathogens to epithelial cells of intestines, which constitute an essential colonization factor during intestinal infections (Sarowska et al., 2019). The detection of a high number of plasmid encoding virulence genes in food, and especially poultry, may serve as a means of transmission of these resistance factors to humans, as observed in this study. The detection of fimbrial genes and a host of other plasmid-encoded genes within the pathogenicity sequences 1–2 among the isolates in this study may signify the potentials of these isolates to cause severe humans infection. Hence, this report will provide valuable reference data that will assist in future investigations of human *Salmonella* infection. In the same vein, the phylogenetic tree obtained from SNPs exhibited diverse clustering of the isolates with an initial one singleton and two clades that subsequently bifurcated. Phylogenetic analysis based on WGS-derived has proven to be a superior cluster resolution method compared to many of the standard subtyping methods (Mohammed and Thapa, 2020; Ford et al., 2021). In order to determine the genetic relatedness among the isolates and infer their evolutionary history based on a genome-wide scale, across the genomes of all *S*. Enteritidis and closely related serovars were identified. Phylogenetic analysis based on the maximum likelihood tree produced strong support for the monophyly of the *Salmonella* isolates with *S.* Enteritidis being the predominant (Figure 3). Within the *S.* Enteritidis isolates from the different sources, a split that delineates two sister lineages, Clade B1 and B2 was observed. At the same time, Clade A consisted of 1 branch occupied by the *E. coli* K12 outgroup, Clade B comprised of the *S*. *bongori* reference strain and the other members of the *S. enterica* serogroup. Sub-clade B2-I which is a branched form the B1 clade, clustered with five of the isolates (S77, S6, S81, S63, and S18) which shared same branches with *S.* Mbandaka, *S.* Weltevreden, *S.* Brancaster, *S.* Kentucky, and *S.* Ohio implying close genetic relationship as determined via the SeqSero serotyping. The major bifurcating clade B2-II, on the other hand, contained one monophyletic clade occupied by *S.* Bandaka and another highly branched sub-clades containing isolates from all the three sources. The fact that samples from poultry, human and food as well as the control *S.* Typhimurium, and *S*. Typhi were located in this well-supported sub-clade may indicate similarity in their gene sequence that can be attributed to convergent evolution due to adaptation to the various sources as earlier reported by Didelot et al. (2007). As deduced from the phylogenetic tree in Figure 3, though the split of clade B is suggestive of allele sharing, possibly via horizontal gene transfer and homologous recombination between the clades, which is a common phenomenon in enteric bacteria (Lawrence and Retchless, 2009; Hall et al., 2020). However, the gene transfer may not have occurred to the extent that divergence between the two clades is eliminated. This is in concordance to divergent lineages observed in *E. coli* following high recombination (Touchon et al., 2009).

## Conclusion

An insight into the genetic profile of enteric *S*. Enteritidis to assess the evolutionary and genetic diversity based on SNPs phylogenomics, as well as understand the virulence and resistance pattern using WGS, was undertaken. The aim was to understand further the genomic diversity existing among *S.* Enteritidis from different host species. The potential transmissibility of genetic elements was evident due to the clustering of multiple isolates from all the three sources. Most of the virulence factors were also detected from the genome of all the isolates across the human, poultry, and food isolates. However, while the genotypic AMR was mostly congruent with the phenotypic profiles of the isolates, slight mismatches were observed with particular reference to gentamicin. This discrepancy was attributed to their silent nature of the *aac(6′)-ly* gene whose transcription occurs intermittently. As a whole, the WGS analysis produced a comprehensive understanding of the resistance profile of S. Enteritidis and the ability of the bacteria to adapt to different host species. However, a more in-depth study, including other *Salmonella* serovars, will be of immense benefit to the understanding of the adaptability and survival mechanism in *Salmonella* serovars.

## Limitation of the Study

This investigation was not able to ascertain the geographical details of the various samples/sources because the isolates were donated as part of stock cultures covering the study period by the agencies involved with the Malaysian National Antimicrobial Resistance Surveillance Program.

## Data Availability Statement

The datasets presented in this study can be found in online repositories. The names of the repository/repositories and accession number(s) can be found in the article/Supplementary Material.

## Author Contributions

ZZ, LH, SAH, and NA: conceptualization. ZS, NMS, and RA: methodology. ZZ, NMS, and BG: software sequence analysis and interpretation. ZZ and BG: writing—original draft preparation. ZZ, BG, LH, NA, SAH, ZS, NMS, and RA: writing—review and editing. ZZ: funding acquisition. ZZ and LH: supervision.

## Conflict of Interest

The authors declare that the research was conducted in the absence of any commercial or financial relationships that could be construed as a potential conflict of interest.

## Publisher’s Note

All claims expressed in this article are solely those of the authors and do not necessarily represent those of their affiliated organizations, or those of the publisher, the editors and the reviewers. Any product that may be evaluated in this article, or claim that may be made by its manufacturer, is not guaranteed or endorsed by the publisher.
